# The Columbian Dental Congress

**Published:** 1892-11

**Authors:** 


### EDITORIAL.

### The Columbian Dental Congress.

The Executive Committee of the Columbian Dental Congress met in Chicago, October 22d, the chief object of which was the election of general officers for the Congress. According to a resolution passed at the previous meeting of the committee the election of officers was fixed for eleven o'clock on the day of this meeting. The Chairman directed this order of business to be taken up. After balloting the following persons were found to be elected, viz.:

For President: Dr. L. D. Shepard, of Boston, Mass.; For Vice Presidents: Drs. W. W. H. Thackston, of Farmville, Va.; Henry W. Morgan, Nashville, Tenn.; A. L. Northrop, New York City, N. Y.; W. W. Allport, Chicago, Ill.; W. O. Kulp, Davenport, Iowa; C. S. Stockton, Newark, N. J.; E. T. Darby, Philadelphia, Pa.; H. J. McKellops, St. Louis, Mo.; J. H. Hatch, San Francisco, Cal.; J. B. Patrick, Charleston, S. C.; John C. Storey, Dallas, Texas.

Several additional persons were appointed upon the various committees.

A scheme or programme for the business of the Congress was outlined, which we will endeavor to give when it is completed, perhaps in the next number of the Register.

